# Factors Influencing Emergence Timing Patterns of Long‐Tailed Bats in Exotic and Native Forest in New Zealand

**DOI:** 10.1002/ece3.70531

**Published:** 2025-02-02

**Authors:** Bonnie Feng, Kerry M. Borkin, Colin F. J. O'Donnell, Joanne M. Monks

**Affiliations:** ^1^ Department of Zoology University of Otago Dunedin New Zealand; ^2^ Department of Conservation Fauna Science, Terrestrial Biodiversity Unit Taupō New Zealand; ^3^ Department of Conservation Fauna Science, Terrestrial Biodiversity Unit Christchurch New Zealand

**Keywords:** behaviour, *Chalinolobus*, conservation, roosting, tree‐felling, Vespertilionidae

## Abstract

Understanding temporal behavioural patterns in animals can be crucial to their conservation management. Emergence timing in bats, that is, the decision on when to depart day‐roosts for foraging, is one such example and is well studied in Northern Hemisphere bats. The emergence timing of New Zealand long‐tailed bats (*Chalinolobus tuberculatus*) is not yet fully understood, including when and where they may be vulnerable to threats. We investigated factors influencing long‐tailed bat emergence timing in the Kinleith Forest (exotic plantation, 38° S) and the Eglinton Valley (native beech forest, 45° S). We recorded emergence times during late pregnancy through post‐lactation (October to March), to determine whether the month, temperature at sunset, tree density, cloud cover, presence of rain and the number of bats within a roost influenced emergence timing. Most long‐tailed bats emerged after sunset in the Kinleith Forest, whilst 80% of first emerging bats departed before sunset in the Eglinton Valley where nights are much shorter in summer, reducing foraging time. Month, temperature at sunset, and roost population size were the most important predictors of emergence timing at both sites. Long‐tailed bats in the Kinleith Forest also emerged earlier as tree density increased, a pattern potentially associated with predator defence. The factors influencing long‐tailed bat emergence timing are likely context dependent, namely latitude and habitat structure, which has implications for roost protection protocols, timing of bat surveys and interpretation of bat acoustic monitoring data.

## Graphical abstract

Factors that impact on emergence timing of long‐tailed bats differ between an exotic and natural forest, and are dependent on the latitude and habitat structure the colony resides within. We recommend updating current conservation practices, including roost protection protocols and timing of bat surveys.

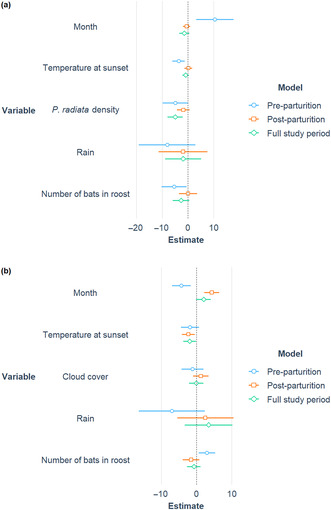

## 1 Introduction

Bats must decide when to emerge from their roost daily. They seek to optimise foraging gain (‘profit’) by accounting for the trade‐off between their ability to feed, their energetic costs, peak prey availability and predation risk in order to survive (Russo, Cistrone, and Jones 2007). Emerge too late and they may miss the peak prey availability, or too early and risk predation. Jones and Rydell (1994) suggest that foraging strategy and predation risk were most important at determining broader patterns of emergence timing for many species, but others have suggested that abiotic factors such as temperature and canopy structure are most likely to influence emergence times because of their effect on bat prey availability (invertebrates) (O'Donnell 2000; Taylor 1963) and protection from predators (Russo, Cistrone, and Jones 2007), respectively.

Emergence timing is also driven by energetic costs largely set by phenological and physiological constraints, the effects of the cost of flight and the need to maintain a high metabolic rate when pregnant, lactating or during spermatogenesis (Racey and Speakman 1987; Racey and Tam 1974). As an in utero foetus grows, for some species, the increasing load increases the energetic costs of flight for their mothers and decreases their manoeuvrability and ability to evade predators, so emergence becomes later (Duvergé et al. 2000; Russo, Cistrone, and Jones 2007). High energetic costs of lactation, and risks of dehydration, are reflected in lactating females emerging earlier as their dependent young grew (Anthony and Kunz 1977; Duvergé et al. 2000; Kurta, Johnson, and Kunz 1987; McLean and Speakman 1999). Young greater horseshoe bats (*Rhinolophus ferrumequinum*), for whom flight is new and costly, at first emerged later than their parents/other adults, but then began emerging earlier as their flying improved (Duvergé et al. 2000).

Light levels and predation risk at the roost site also likely impact emergence timing decisions. Canopy structure and distance from the forest edge have been found to affect when bats of different species emerge from their roosts (Arndt et al. 2018; Funakoshi and Uchida 1978; Russo, Cistrone, and Jones 2007; Rydell, Entwistle, and Racey 1996). Some species of bats may assess predation risk of vision‐dependent predators to be lower in relatively cluttered, high canopy cover areas, so they emerge earlier, allowing for prolonged feeding durations (Russo, Cistrone, and Jones 2007; Rydell, Entwistle, and Racey 1996).

Temperature may influence emergence indirectly because of its effect on the prey of insectivorous bats (Anthony, Stack, and Kunz 1981; Rydell 1989), but this has not be found for all species (Viele, Kurta, and Kath 2002). Cloud coverage and precipitation level effects on emergence timing also vary (Arndt et al. 2018; Funakoshi and Uchida 1978; Swift 1980; Thomas and Jacobs 2013). Cloud coverage likely offers similar benefits to canopy cover as it decreases a predator's visibility by blocking moonlight (Arndt et al. 2018; Welbergen 2006). Rain can impede flight and be energetically costly (Voigt et al. 2011), so bats may alter their emergence time to avoid heavy rain or showers (Doty, Gonsalves, and Law 2019; Funakoshi and Uchida 1978; Shiel and Fairley 1999); however, this is not a pattern found in all bat species (Swift 1980). Some bat species may also emerge earlier after periods of low prey availability caused by inclement weather (Frick et al. 2012).

When the number of bats within a roost is greater, bats often begin to emerge earlier (Arndt et al. 2018; Warren and Witter 2002), but because of the large number of individuals queueing to leave (Hristov et al. 2010) median time of emergence may be later (Avery 1986; McAney and Fairley 1988). Again, this is not a pattern found for all species (Jones 1995; Russo, Cistrone, and Jones 2007). The lack of a universal pattern for all these influences means that the effects of factors on a species emergence pattern must be examined for each species separately for them to be meaningful.

Long‐tailed bats (*Chalinolobus tuberculatus*, pekapeka) are one of two extant endemic New Zealand bat species. They are a nocturnal insectivorous temperate species belonging to the family Vespertilionidae and are classified as Nationally Critical under the New Zealand Threat Classification System (O'Donnell et al. 2023) and Critically Endangered by the International Union for Conservation of Nature (O'Donnell 2021). Long‐tailed bats typically roost within tree cavities and under peeling bark (Sedgeley and O'Donnell 1999a) but some also roost within caves (O'Donnell 2002a). As bats are New Zealand's only native terrestrial mammal, their only suspected natural predators are birds. These include the nocturnal morepork (*Ninox novaeseelandiae*) (Dwyer 1962), extinct laughing owl (*Ninox albifacies*) (Worthy and Holdaway 2002), diurnal Australasian harrier (*Circus approximans*) and New Zealand falcon (*Falco novaeseelandiae*) (Sedgeley and O'Donnell 1999b). Research into long‐tailed bats' emergence timing patterns has been limited to assessments of the effect of weather and moonlight intensity to date. Minimum overnight temperature was a strong determinant of whether or not long‐tailed bats emerged at all (O'Donnell 2000). Griffiths (1996) found that temperature at sunset was the only significant predictor of emergence timing of long‐tailed bats in South Canterbury (44°11 S, 170°01 E). Nocturnal activity of long‐tailed bats in Hawke's Bay (39°27 S, 176°50 E) began earlier under low mean moonlight intensity, likely due to prey insects avoiding emergence under high moonlight intensities (Gillingham 1996).

Visual emergence surveys (emergence or roost watches) are used to understand roost use (O'Donnell and Sedgeley 1999) and population size (Kunz et al. 2009), identify maternity roosts (Mitchell‐Jones 2004), and ensure bats are not present in roosts when trees or buildings are modified (Collins et al. 2016; Scheunert, Zahn, and Kiefer 2010). The reliability of these measures is dependent on understanding emergence patterns and their drivers. Bats (Chiroptera) are members of a highly diverse order where a one‐size‐fits‐all conservation method does not exist; each species is unique and possesses different behaviours, warranting species‐specific management plans (Fenton 1997). Detailed understanding of long‐tailed bat emergence therefore represents a gap in the knowledge that is crucial for informing conservation practises such as tree‐felling protocols. These protocols aim to identify and protect potential roost trees by performing emergence watches; understanding the correct time to carry these out is essential.

Based on global literature, we predicted long‐tailed bats would emerge earlier:

1. as the summer progresses and bats shift from pre‐parturition towards post‐lactation period (Lee and McCracken 2001; Russo, Cistrone, and Jones 2007) and more specifically, as lactation advances (Duvergé et al. 2000; Russo, Cistrone, and Jones 2007),
2. with increasing sunset temperature (Anthony, Stack, and Kunz 1981; Griffiths 1996; Rydell 1989),
3. under higher tree density and/or cloud cover (Arndt et al. 2018; Funakoshi and Uchida 1978; Russo, Cistrone, and Jones 2007; Rydell, Entwistle, and Racey 1996),
4. with decreased level of precipitation (Funakoshi and Uchida 1978; Shiel and Fairley 1999) and
5. with decreasing roost population size (Arndt et al. 2018; Warren and Witter 2002).

Moreover, bats were predicted to continuously emerge *later* as gestation progresses (Duvergé et al. 2000; Russo, Cistrone, and Jones 2007).

## 2 Materials and Methods

### 2.1 Study Sites

**FIGURE 1:**
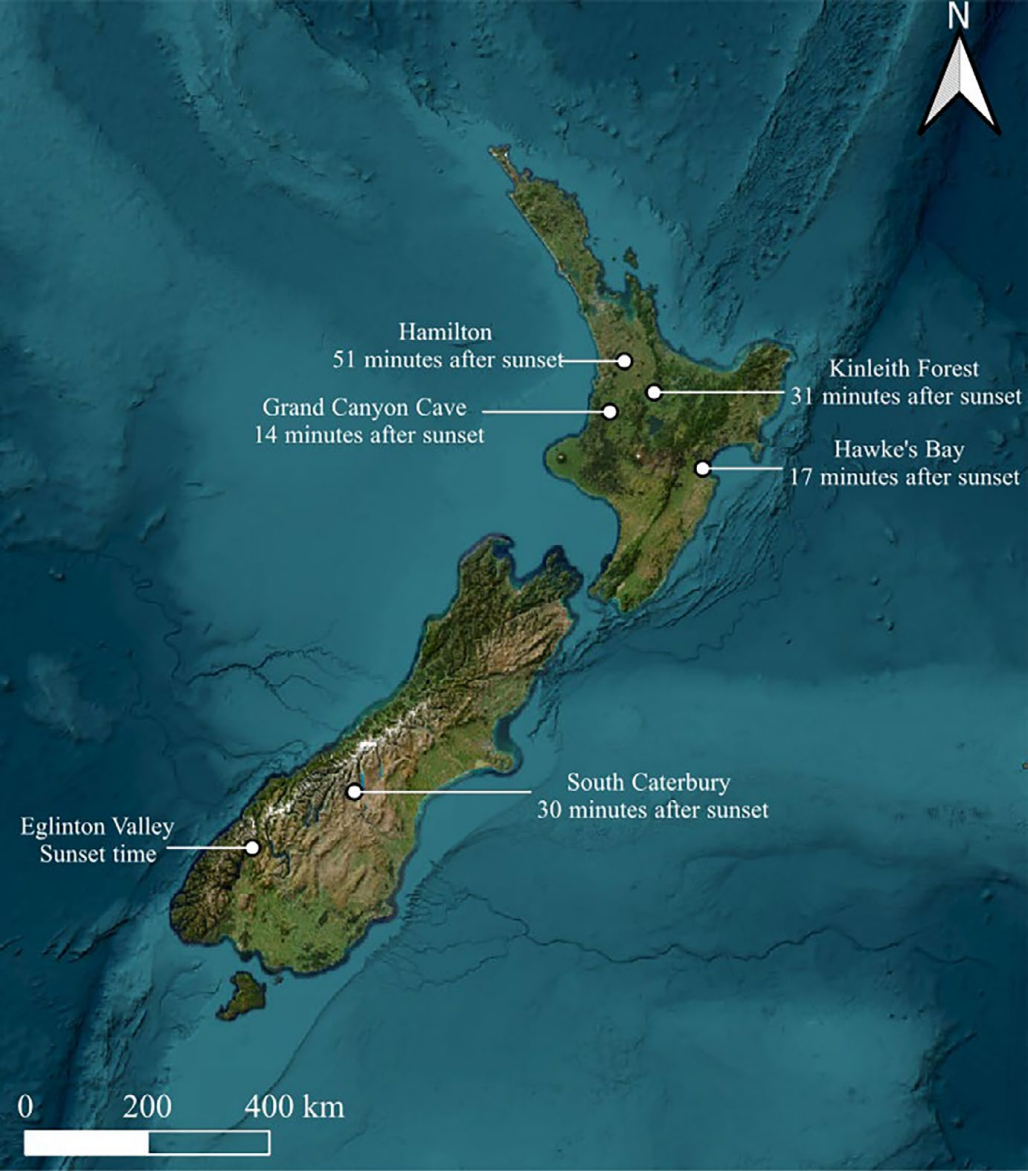
Summary of mean emergence times of long‐tailed bat (*Chalinolobus tuberculatus*) colonies across New Zealand. Mean emergence times at Hamilton, Grand Canyon Cave, Hawke's Bay and South Canterbury colonies were extracted from O'Donnell and Borkin (2021). Map was created with QGIS.

Long‐tailed bat emergence data were collected from the Kinleith Forest, a large exotic plantation forest primarily composed of plots with uniformly planted pine trees (*Pinus radiata*) of similar size, in the Waikato Region of the North Island (38°17′ S, 175°53′ E). The site had a mean elevation of 632 m above sea level (a.s.l.) and was 96,652 ha at the time of data collection (Figure 1) over three consecutive austral summers (November to March of 2006–2009).

Emergence timings of long‐tailed bats were also collected from the Eglinton Valley, a native forest (elevation of 300 m (a.s.l.)) mainly comprising of native red beech (*Fuscospora fusca*), mountain beech (*F. cliffortioides*) and silver beech (*Lophozonia menziesii*) in Fiordland National Park (44°58′ S, 168°01′ E) (Figure 1). The forest was old and complex with multiple strata and spreading crowns. Data were collected over three consecutive austral summers (October to March of 1993–1996) and supplemented with observations from January and February 2022.

### 2.2 Data Collection

Roost watches were undertaken in relatively fine weather (not in heavy rain) and where roosts could be safely accessed by observers in the dark. These limitations are unlikely to be a large bias as bats do not typically emerge under heavy precipitation (Funakoshi and Uchida 1978; Shiel and Fairley 1999) and those that roost far from the flats of Eglinton Valley are likely to be post‐lactation and therefore solitary (roosts that contain only one bat) (O'Donnell and Sedgeley 1999). In conjunction with visual counts of emerging bats, we used a handheld bat detector to corroborate observations.

#### 2.2.1 Kinleith Forest

Long‐tailed bats were captured and radio‐tagged. Roost trees were located using radio telemetry and emergence watches took place as described by Borkin, O'Donnell, and Parsons (2011). Each individual bat's emergence time stamp was subtracted from the sunset time (from the NZ Nautical Almanac; LINZ (2006, 2007, 2008)) to produce their emergence time relative to sunset, the dependent variable (negative values denote emergences before sunset, positive values denote after). A Vertex III and Transponder T3 (Haglöf Sweden AB) were used to measure distance from the roost tree to determine the number of pine trees planted within a 10 m radius as a measure for tree density. Weather variables (temperature at sunset and level of rainfall in the hour before sunset) were obtained retrospectively from the Athol base weather station situated at the study site through the Rural Fire Protection Authority.

#### 2.2.2 Eglinton Valley

Long‐tailed bats were captured and radio‐tagged. Roost trees were located and confirmed using radio telemetry and emergence watches took place as described in O'Donnell and Sedgeley (1999). Emergence times were recorded and subtracted from sunset times (from the NZ Nautical Almanac; Lamont (1993, 1994, 1995)) to produce their emergence time relative to sunset (dependent variable). Cloud cover was measured by eye at sunset in Oktas (8 representing 100% coverage and 0 as 0%). Rain presence at sunset was recorded as yes (1) or no (0). Temperature at sunset was extracted retrospectively via NIWA (National Institute of Water and Atmospheric Research 2022) recorded by Knob's Flat weather station situated at the study site.

In the 2022 study season, free standing harp traps were set up in known high bat traffic areas to catch suitable lactating female bats for transmitter attachment (Model BD2, Holohil, Canada). To maximise bat attraction, acoustic bat lures (Sussex Autobat 1 and 2, Sussex University, United Kingdom) were attached to some traps. These devices played a synthesised social bat‐call known as ‘Bechsteins2’ (Hill and Greenaway 2005) and were connected to a two‐way ultrasonic speaker (Polaroid 600 Series, Environmental Grade electrostatic transducer). Traps were checked 1 h before sunrise, whereby each bat was processed following methods outlined by O'Donnell and Sedgeley (1999). Roost watches were carried out from 1 h before sunset until 20 min after the last observed bat emergence. Time was noted for each emergence using a phone application ‘TimeStamp’ (version 0.4.1; developed by m_c8bit). Sunset for each roost on each roost watch night was calculated via Global Monitoring Laboratory (2009) using roost latitude–longitude coordinates (geographic coordinate system). Minutes from sunset were calculated as above.

### 2.3 Data Analysis

Solitary bats from the Eglinton Valley frequently enter torpor due to low temperatures even in summer, so we excluded these from our analyses of emergence data (McNab and O'Donnell 2018). Roosting communally increases the overall temperature of the roost, so bats are less likely to enter torpor and emerge from their roosts (Webb 1999). Solitary bats were not excluded from the Kinleith Forest dataset as bats are more likely to be active there each night as temperatures are on average higher, and the types of roosts most commonly used—under loose bark—means that they cannot house large numbers (Sedgeley and O'Donnell 1999b).

To understand factors influencing emergence time, linear mixed models were fitted with restricted maximum likelihood using the package ‘lmerTest’ (Kuznetsova, Brockhoff, and Christensen 2017). As bats in the same roost are not independent of each other, true observations were at the roost level for both study sites. As some roosts were visited more than once (36%) across both sites over the study period, a visitation number was assigned to each roost watch and a nested random effect was added (visit number nested within each unique roost) in all analyses. This accounts for the non‐independence of individual bats in the same roost, allowing us to use each individual bat's emergence as the dependent variable. As different roosts watched on the same day did not occur often, the observation date was not added as a random effect. We used the Satterthwaite method (see Giesbrecht and Burns (1985)) to determine degrees of freedom and *p* values as this method gives sufficiently accurate Type I error rates at the 0.05 level, even with smaller sample sizes (Luke 2017). As long‐tailed bat maternity roosting aggregations are outnumbered by reproductive females (O'Donnell and Sedgeley 1999) and may not respond to the variables the same way at different points in the summer, the datasets were split into pre‐parturition (October to December) and post‐parturition (January to March) (O'Donnell 2002b) and analysed separately as well as together. Reproductive seasonality was measured in months whereby the number one was assigned to the first month data collection took place each season, two to the second month and so on, and was treated as a continuous variable. Before model fitting, diagnostic checks were implemented to ensure normality in the emergence time of each individual bat relative to sunset (minutes) dependent variable for each subset. For the Kinleith datasets (full, pre‐parturition and post‐parturition), we included month, temperature at sunset, rain, pine tree density and number of bats in roost as the predictor variables. For the Eglinton datasets, we included month, temperature at sunset, rain, cloud cover and number of bats in roost. We found each observation's influence using package ‘influence.ME’ (Nieuwenhuis, Te Grotenhuis, and Pelzer 2012) and then calculated the Cook's Distance of these values. No datapoints surpassed the 4/*N* threshold (where *N* represents the true number of replicates), and thus none were deemed influential.

All datasets followed a normal distribution during diagnostic checks, and all models visually satisfied model checking requirements. Multicollinearity was not detected as variance inflation factors of all predictors from all models were between 1 and 2. No outliers were detected during model checking.

Regression coefficient plots were constructed on the standardised scale for each data subset to visualise variable estimates and their standardised confidence intervals (CIs) using the ‘jtools’ package (Long 2022). Coefficients and their CIs were standardised for appropriate comparison of relative importance as the predictors are on the same scale. The binary variable rain presence was coded as a 0/1 dummy variable for appropriate standardisation; care was taken to ensure all variable point estimates and CIs were interpreted on the natural scale rather than the visualised standardised scale, with all other effects held fixed. Effects from pre‐parturition and post‐parturition subsets were interpreted only if the effect over the full season was non‐significant at the type I error rate of 0.05. Due to their arbitrary definition, we do not report *p*‐values; we instead report *t*‐values and their associated degrees of freedom being. Reported 95% coefficient CIs were calculated following the Wald method using Satterthwaite‐derived degrees of freedom via package ‘parameters’ (Lüdecke et al. 2020). All statistical analyses were carried out in R version 4.0.2 (RStudio Team 2021).

## 3 Results

In total, we observed 324 bats emerging from 30 unique roosts over three breeding seasons in the Kinleith Forest, and 4948 bats emerging from 103 unique roosts over four breeding seasons in the Eglinton Valley. Most observed roosts in the Kinleith forest were under peeling bark, whereas in the Eglinton Valley, the observed roosts were mostly tree cavities. The median number of bats occupying the same roost in the Kinleith Forest was 1 (range = 1 to 20), whereas the median number of bats seen emerging from the same roost in the Eglinton Valley was 32 (range = 2 to 122). Of the 134 independent roost watches carried out in the Kinleith Forest, 94 were solitary roosts (70%) whereas 45% were solitary (115 out of 259) in the Eglinton Valley. Solitary observations from the Eglinton Valley were omitted from analyses reported below due to the probability this resulted from temperature induced torpor (see Methods). As solitary roosts were found proportionally more in October and March, these most likely represent the males that have not yet become active in the breeding season and females that have completed the breeding season, respectively (O'Donnell and Sedgeley 1999).

During the study period, temperatures at sunset ranged from 10°C to 21.3°C (mean = 16.2°C) in Kinleith Forest and 7.1°C–29.5°C (mean = 14.8°C) in Eglinton Valley on nights that bats emerged. In Kinleith Forest, the number of planted *P. radiata* surrounding the roost tree within a 10 m radius (*P. radiata* density) ranged between 5 and 23 (median = 11) and the precipitation in the hour before sunset ranged between 0 and 1.02 mm (median = 0 mm). As this is classified as light rain (Meteorological Service of Canada 2013), the data were converted into binary code (1 was assigned to nights that rained in the hour before sunset and 0 otherwise). Thus, it rained in the hour before sunset for seven independent roost watches (5.5%) in Kinleith Forest. In Eglinton Valley, it rained at sunset for 17 independent roost watches (11.8%) and the mean cloud cover was 2.5 oktas (32%).

### 3.1 Variation of Emergence Timing

**FIGURE 2:**
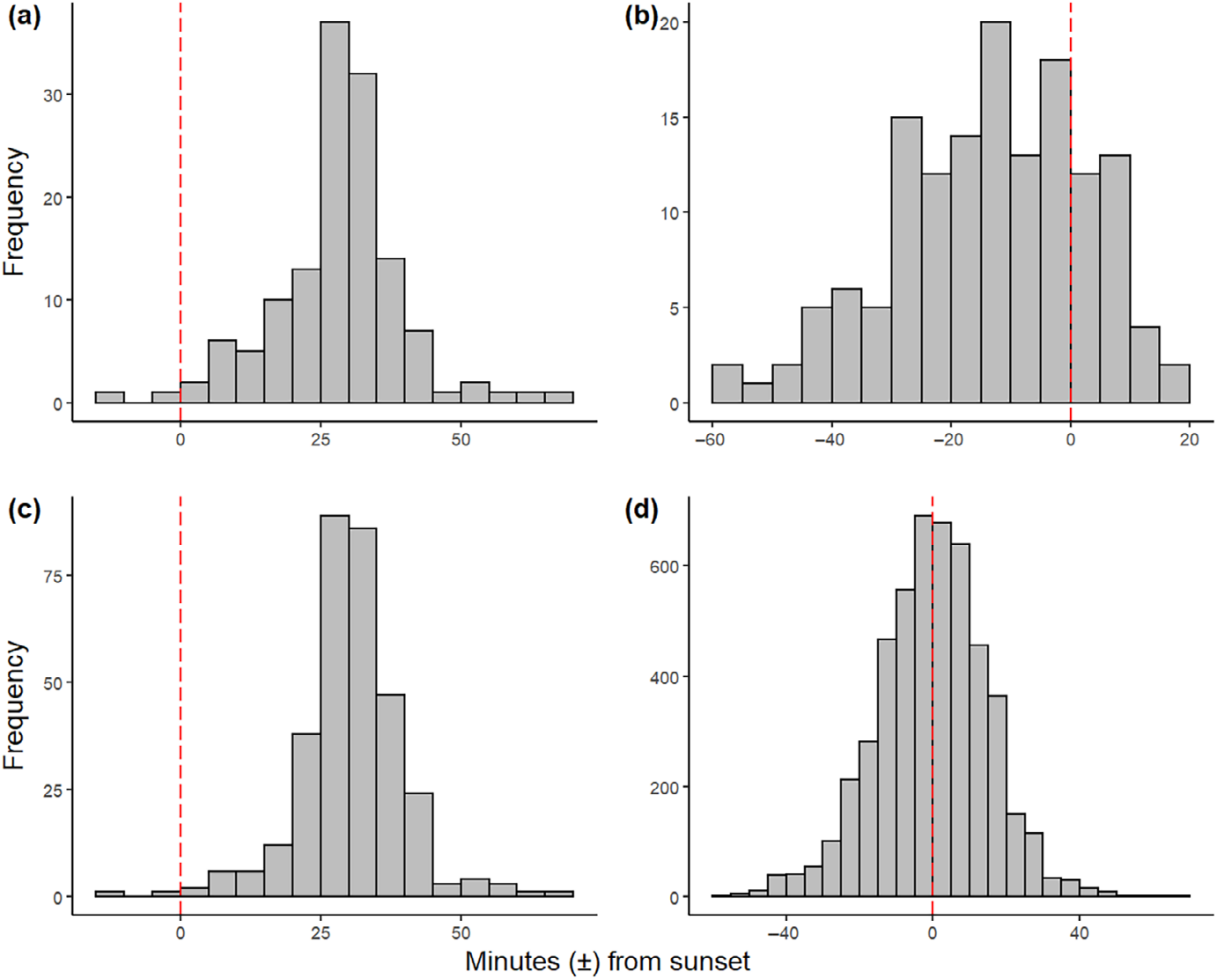
Distribution of emergence times of long‐tailed bats relative to sunset (zero; red dotted line), with negative numbers being minutes before sunset and positive numbers being minutes after sunset for two forests in New Zealand during the summer (October to March). In Kinleith Forest (a) emergence start times (*n* = 134) and (c) individual emergence times (*n* = 324) were predominantly after sunset. In the Eglinton Valley (b) emergence start times (*n* = 144) were predominantly before sunset and (d) individual emergence times (*n* = 4948*) peaked during sunset. *Solitary roosting individuals excluded (*n* = 115).

In total, we included data from 134 independent roost watches for analysis in the Kinleith dataset and 144 in the Eglinton dataset. Bats were observed emerging in Kinleith Forest from 14 min before sunset to 68 min after sunset and in Eglinton Valley from 56 min before sunset to 66 min after sunset. Most first emergences occurred before sunset in the Eglinton Valley and after sunset in Kinleith Forest (Figure 2a,b). Most emergence times from Kinleith Forest were centred on 30 min after sunset (Figure 2c) and on sunset time (0 min) in Eglinton Valley (Figure 2d).

#### 3.1.1 Timing of Bat Emergence in the Kinleith Forest

**FIGURE 3:**
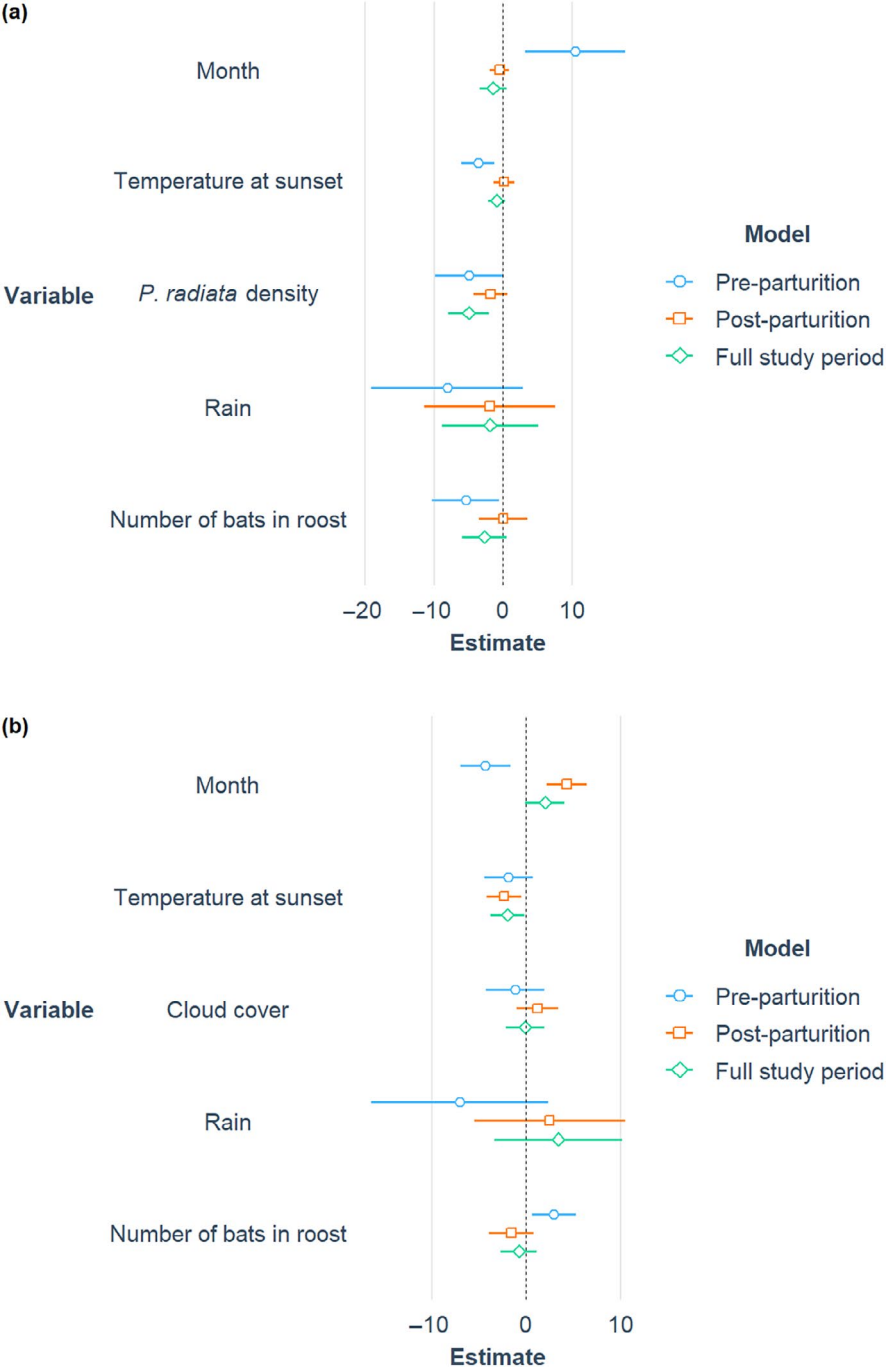
Visualisation of the abiotic and biotic influences on the variation in long‐tailed bat emergence timing at (a) the Kinleith Forest (*N* = 45 during pre‐parturition (November to December), *N* = 89 during post‐parturition (January to March)) and (b) the Eglinton Valley (*N* = 45 during pre‐parturition (October to December), *N* = 99 during post‐parturition (January to March)) at the roost level. Regression coefficients and their constructed 95% confidence intervals were produced from linear mixed models and then standardised. Intervals that are wholly above zero indicate a positive relationship and intervals that lie wholly below zero indicate a negative relationship. Estimates whose 95% confidence interval overlaps zero are not considered to be statistically significant.

**FIGURE 4:**
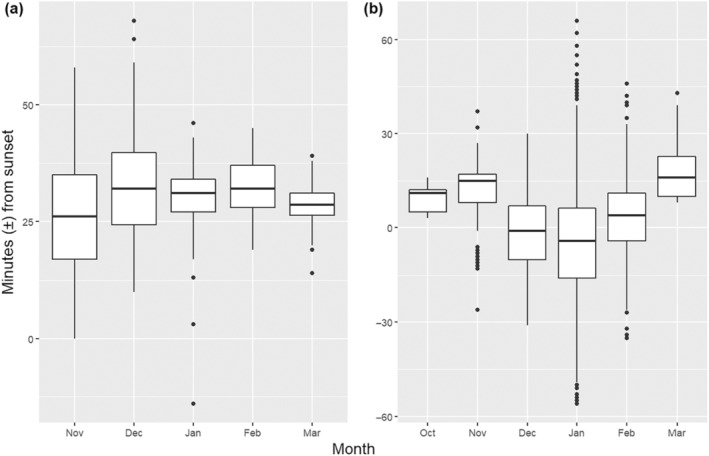
Trend in individual emergence times of long‐tailed bats relative to sunset in the (a) Kinleith Forest from 324 emergences observed between November and March of 2006–2009, and (b) Eglinton Valley from 4948 emergences observed during October to March of 1993–1996 and January to February 2022.

**FIGURE 5:**
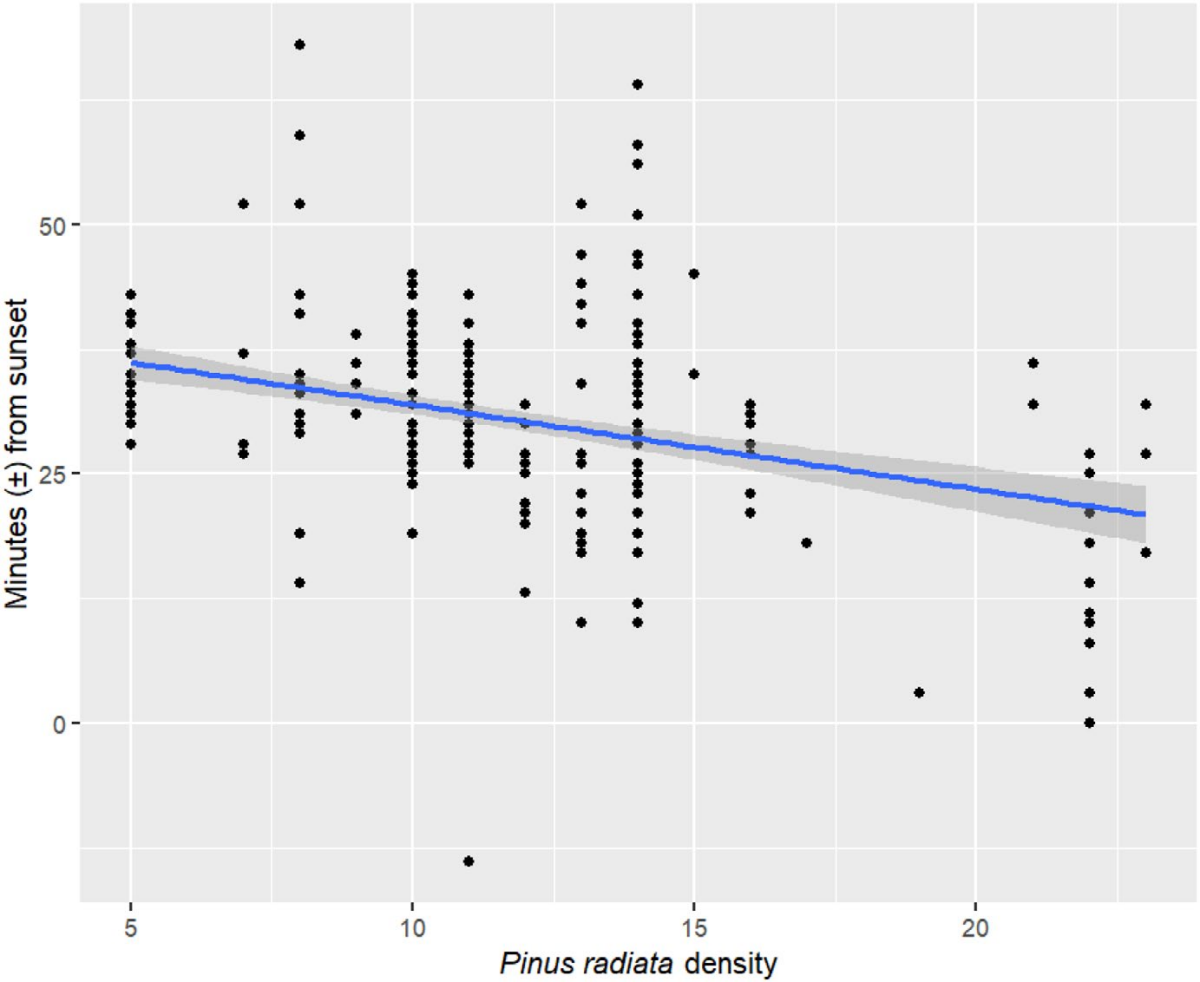
The relationship between individual emergence timing of long‐tailed bats relative to sunset and tree density (number of *P. radiata* in a 10 m radius from the roost tree) in the Kinleith Forest between November and March of 2006–2009.

Although there was no overall effect of month across the full study period and during post‐parturition, bats emerged later as pre‐parturition progressed (coefficient = 10.5, i.e., 10.5 min later per month, CI = 3.0–17.9, *t*_35.4_ = 2.826) (Figures 3a and 4a). Temperature at sunset had a significant negative relationship with emergence timing only during pre‐parturition such that the warmer it was at sunset, the earlier bats emerged (coef. = −1.8, i.e., 1.8 min earlier per 1°C increase in temperature, CI = −2.9 to −0.6, *t*_46.3_ = −2.997). The higher the tree density in the Kinleith Forest, the earlier bats emerged across the full study period (coef. = −1.2, CI = −2.0 to −0.5, *t*_26.2_ = −3.285) (Figures 3a and 5). Rain presence in the hour before sunset was not a significant predictor for emergence during any period (Figure 3a). During pre‐parturition, the more bats in the roost, the earlier bats emerged (coef. = −2.2, CI = −4.3 to −0.1, *t*_19.6_ = −2.188). On the standardised scale, the most important drivers of emergence timing during pre‐parturition appeared to be the change in months, followed by tree density, roost population size and temperature at sunset (Figure 3a).

#### 3.1.2 Timing of Bat Emergences in the Eglinton Valley

**FIGURE 6:**
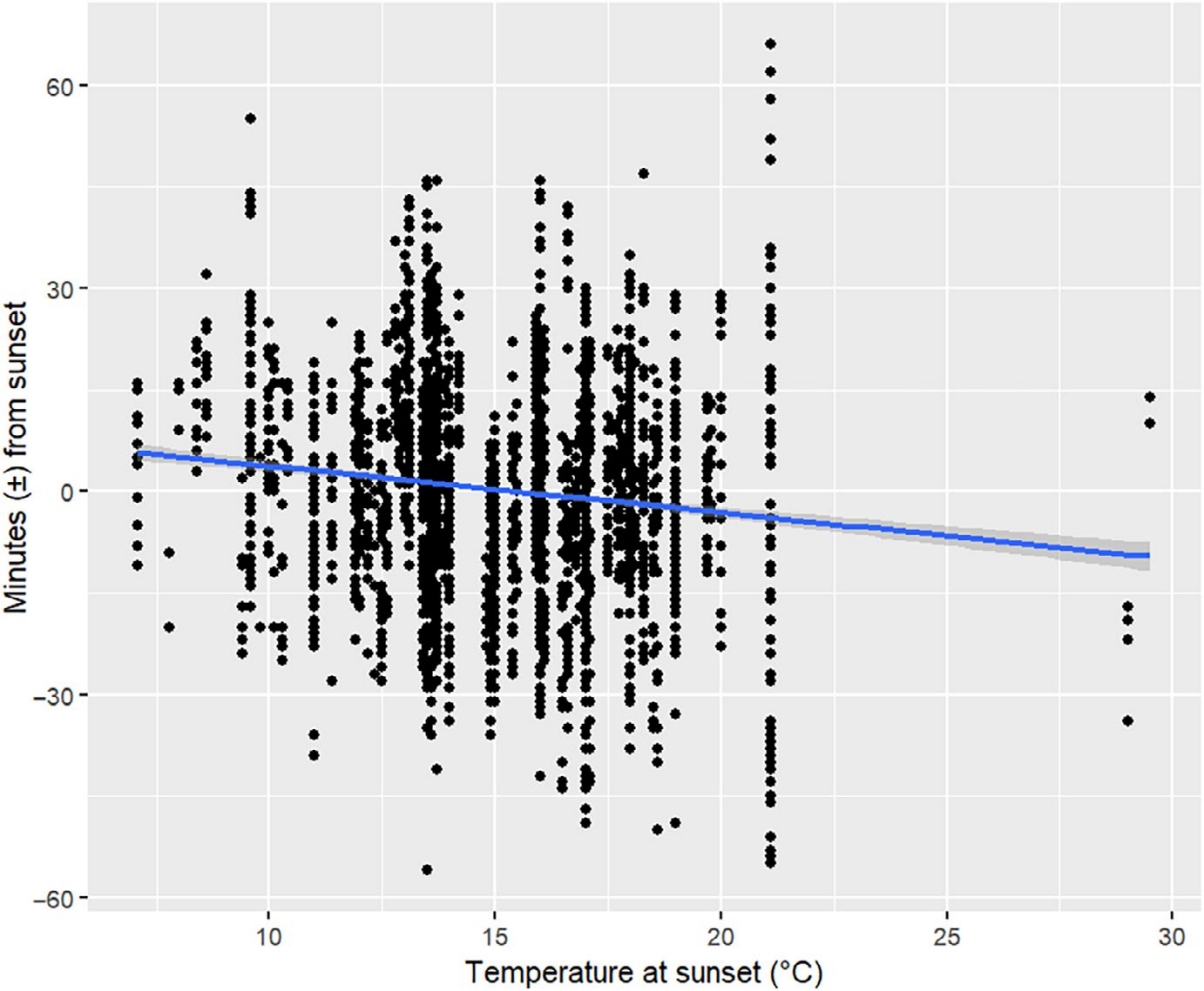
The effect of temperature at sunset on individual emergence timing of long‐tailed bats relative to sunset in the Eglinton Valley during October to March of 1993–1996 and January to February 2022.

Bats emerged *earlier* during pre‐parturition (coef. = −7.9, CI = −13.0 to −2.8, *t*_24_ = −3.187) and *later* during post‐parturition (coef. = 8.5, CI = 4.2 to 12.8, *t*_66_ = 3.963) (Figures 3b and 4b) contrary to our predictions. As temperature at sunset increased, bats emerged earlier across the full study period (coef. = −0.7, CI = −1.3 to −0.1, *t*_158.0_ = −2.167) (Figures 3b and 6). Cloud cover at sunset and presence of rain at sunset was not a significant predictor for emergence timing across the full study period nor during pre‐parturition and post‐parturition (Figure 3b). Although the relationship between the total number of bats seen emerging from the roost and emergence timing was not significant over the full study period nor during post‐parturition (Figure 3b), it was significantly positive during pre‐parturition whereby the more bats there were occupying the roost, the later bats emerged (coef. = 0.2, CI = 0.1–0.3, *t*_39_ = 2.459). On the standardised scale, reproductive seasonality (month) and roost population size were the most important predictors followed by temperature at sunset (Figure 3b).

## 4 Discussion

Long‐tailed bats in the Eglinton Valley emerged consistently earlier than those in the Kinleith Forest, and the factors driving emergence timing differed between the two sites. Reproductive seasonality was an important predictor at both sites during pre‐parturition but affected emergence timing inversely. This opposing effect at the two sites was also seen in the number of bats occupying the roost during pre‐parturition. Weather variables were not the key drivers of emergence in this study; temperature had a small effect and rain had no effect at both study sites. Tree density and cloud cover acted as proxies for drivers of anti‐predator behaviour in the Kinleith Forest and Eglinton Valley, respectively; however, only tree density was detected to influence emergence timing. As this relationship is not fully understood, more research is required.

Our study of two long‐tailed bat populations enabled us to examine influences on nightly emergence timing at maternity roosts over the summer period when bats were heavily pregnant (pre‐parturition) or lactating and newly volant (post‐parturition). During pre‐parturition, emergence timing was more sensitive to environmental factors in the Kinleith Forest than during times when bats were lactating and newly volant, likely driven by female reproductive status. Several species of bats are active for shorter periods when heavily pregnant than when lactating (Anthony, Stack, and Kunz 1981; Swift 1980) including long‐tailed bats (O'Donnell 2002b). Flight likely has high energetic costs for pregnant bats, whilst the requirement to maintain relatively high metabolic levels for foetal development means they are less likely to enter torpor. Consequently, we expect that pregnant bats are under more pressure to emerge at an optimal time to balance these needs. We note that the physiological demands within pregnancy in long‐tailed bats are quite variable in comparison to post‐parturition (O'Donnell 2002b). Thus, we expect that female bats in earlier pregnancy may respond differently to environmental cues compared to those at late pregnancy, explaining the large CI seen for effects during pre‐parturition.

We expected that long‐tailed bats would emerge earlier as bats progressed from pre‐parturition towards post‐lactation. We only observed this pattern of earlier emergence as the summer progressed during pre‐parturition in the Kinleith Forest. In the Eglinton Valley, we observed long‐tailed bats emerging earlier as pregnancy advanced and then later as lactation advanced towards weaning. We suspect that during post‐parturition, emergence timing may be influenced by the late emergence of juvenile bats (Petrželková and Zukal 2001), resulting in a non‐significant effect when looking at the summer as a whole. The result may have been different if we were able to exclude juvenile bats from our analyses. Juvenile bats are relatively inexperienced poor fliers and tend to emerge much later likely to avoid predation (Petrželková and Zukal 2001). We suspect that juvenile long‐tailed bats were weaned relatively quickly, with 65% of captured females classified as post‐lactating in the first week of February (O'Donnell 2002c), allowing later emergence by mothers as the energetic cost of lactation subsides as seen in Mexican free‐tailed bats (*Tadarida brasiliensis*) (Lee and McCracken 2001; Lima and O'Keefe 2013). It is noted that in some species, juveniles emerge earlier than adults to keep up with nutritional needs once weaned (Lee and McCracken 2001; Reichard et al. 2009), or as their flying abilities improve (Duvergé et al. 2000). The counteracting patterns of emergence timing between age classes may also explain the non‐significant effect of changing months in our study across the whole breeding season.

The vast majority of current literature shows bats emerge later as pregnancy progresses and gradually emerge earlier as they progress from lactation towards weaning (Duvergé et al. 2000; Jones 1995; Russo, Cistrone, and Jones 2007). However, Arndt et al. (2018) found that first emerging Indiana bats (*Myotis sodalis*) departed earlier as pregnancy progressed, associating this to the variation of prey availability, predator behaviour and abiotic factors. During one summer, Petrželková and Zukal (2001) found that serotine bats (*Eptesicus serotinus*) emerged earlier during the post‐parturition period than pre‐parturition period; however, the opposite was true in the previous summer, with the authors citing annual differences in prey availability. Invertebrate availability increased and peaked during post‐parturition in the Eglinton Valley (O'Donnell 2000); thus, lactating long‐tailed bats may not need to emerge progressively earlier during post‐parturition to feed their growing young (Duvergé et al. 2000).

We found that the warmer the temperature at sunset, the earlier long‐tailed bats emerged, consistent with results for other temperate bat species (Anthony, Stack, and Kunz 1981; Griffiths 1996; Rydell 1989). Previous research found that long‐tailed bats emerged earlier on nights when the sunset temperature was higher (Griffiths 1996). Temperature can guide bats in matching nightly foraging activity with peak invertebrate activity, and they may take longer to arouse from torpor on colder days and thus leave the roost later (O'Shea and Vaughan 1977). The presence of light rain did not influence emergence timing at both study sites. Although emergence timing in many bat species is not affected by light rain, some species emerge after or between bouts of heavy rain (Entwistle, Racey, and Speakman 1996; Erkert 1982; Shiel and Fairley 1999; Weinbeer, Meyer, and Kalko 2006). As we did not monitor emergences under heavy rain, we could not test this hypothesis, highlighting an opportunity for further research.

We observed that bats in Kinleith Forest rarely emerged before sunset, whereas the majority (80%) of first bats emerged from roosts before sunset in the Eglinton Valley. These two populations occupy habitats which vastly differ in vegetation structure, roost types used and latitude. These may all have implications for emergence.

We suspect that the higher latitude (44°58′ S) of the Eglinton Valley compared to the Kinleith Forest (38°17′ S) could help explain this difference. Rydell, Entwistle, and Racey (1996) suggested that bats living in higher latitudes benefit more by emerging early compared to those at lower latitudes. At high latitudes, the nights are generally shorter and cooler, so invertebrates are less active and their abundance drops more quickly on shorter nights (O'Donnell 2000; Rautenbach, Kemp, and Scholtz 1988; Taylor 1963). Over the summer, nights become shorter towards the summer solstice and longer afterwards. This rate of change is greater at higher latitudes compared to areas of lower latitude. For example, night length of the 2021 summer solstice (22 December) was 8 h and 26 min in the Eglinton Valley, whereas it was 9 h and 15 min in the Kinleith Forest (Global Monitoring Laboratory 2009). Rate of change in night duration is thus greater in the Eglinton Valley moving towards the autumn equinox, perhaps driving the earlier emergence towards the summer solstice and later emergence afterwards at pre‐parturition and post‐parturition, respectively.

Roosts under peeling bark within plantation forests do not support the relatively high temperatures found in roost cavities used by bats in the Eglinton Valley (Borkin and Parsons 2011; Sedgeley 2001), which may contribute to later emergence times in the Kinleith Forest. The effect of roost population was only significant during pre‐parturition at both sites but in opposite directions; an increase of bats in a roost was associated with earlier emergence in the Kinleith Forest, whilst it resulted in later emergence in the Eglinton Valley. Roost population sizes were far smaller within the Kinleith Forest compared to the Eglinton Valley. Bats in smaller group sizes take longer to warm up after daily torpor (Willis and Brigham 2007) and need not grapple to avoid getting stuck in the queue to leave (Hristov et al. 2010). Similar to Avery (1986) and McAney and Fairley (1988), we found that bats in the Eglinton Valley left later as the roost population grew. In the Eglinton Valley, long‐tailed bats may start emergence earlier to compensate for the need to wait for their turn to leave (Feng, unpublished data). Increased roost population size is also associated with higher ambient temperature in roosts (Willis and Brigham 2007), when particularly high, bats increase their rates of evaporative cooling through respiration and through their skin (Reeder and Cowles 1951). This can lead to dehydration (Korine, Zinder, and Arad 1999), forcing earlier emergence. There was not a threshold roost size whereby long‐tailed bats started to emerge earlier (Feng, unpublished data). We noted no relationship between roost population size and emergence time during post‐parturition at both study sites, indicating other factors are more important when juveniles are present.

The Kinleith Forest and the Eglinton Valley are both occupied by morepork. Plantation forests are thought to have high densities of falcon (Seaton 2007) due to increased bird prey abundance, and are known for their importance in supporting native avian predators (Pawson et al. 2010). Therefore, long‐tailed bats at Kinleith Forest may be exhibiting predator defence mechanisms including emerging after sunset and emerging earlier under higher tree densities. Tree density and canopy cover were lower at roost trees compared to available trees in the Eglinton Valley as emerging in more open forest structures may allow better manoeuvring (Sedgeley and O'Donnell 1999a). This can potentially be useful for both predator evasion and feeding efficiency. More research is required around these interpretations.

### 4.1 Conservation Implications

Long‐tailed bats most often roost in the oldest trees available in the landscape (Borkin 2010; Sedgeley and O'Donnell 1999b), and these trees are often targeted during logging operations or for firewood in plantation forests (Borkin, O'Donnell, and Parsons 2011; Sedgeley 2001; Sedgeley and O'Donnell 2004) or removed due to risks to humans. Where trees are felled in bat habitats, current best practice protocols aim to reduce the likelihood of killing or injuring bats in their roost, following strict guidelines to ensure no bats are inside during felling (New Zealand Department of Conservation's Bat Recovery Group 2021). Checking for bat presence involves a step‐by‐step approach at times requiring emergence watches. The guidance recommends emergence watches begin at sunset and continue until it is too dark to see. If no bats are observed during the roost emergence watches, it is concluded that the roost tree of interest is not occupied, and the tree can be felled. As first emergences in the Eglinton Valley occurred consistently *before* sunset, we strongly recommend revising these protocols so that they start earlier to observe all bats leaving roosts there. There may be other colonies where these protocols do not capture all emergence times because these vary according to location (Figure 1) and time of year. We recommend using high quality of infrared or thermal cameras particularly at lower latitudes where bats likely emerge after dark, although this may vary by habitat types that trigger other pressures on emergence timings. Our findings can also be used to improve monitoring for conservation purposes as they aid researchers in timing surveys for maximum detectability.

## Author Contributions

**Bonnie Feng:** conceptualization (supporting), data curation (supporting), formal analysis (lead), methodology (supporting), project administration (lead), writing – original draft (lead), writing – review and editing (equal). **Kerry M. Borkin:** conceptualization (lead), data curation (lead), funding acquisition (lead), methodology (lead), supervision (supporting), writing – original draft (supporting), writing – review and editing (equal). **Colin F. J. O'Donnell:** conceptualization (supporting), data curation (lead), funding acquisition (lead), methodology (lead), resources (supporting), supervision (supporting), writing – review and editing (supporting). **Joanne M. Monks:** conceptualization (supporting), formal analysis (supporting), methodology (supporting), project administration (lead), resources (supporting), supervision (lead), writing – original draft (supporting), writing – review and editing (equal).

## Conflicts of Interest

The authors declare no conflicts of interest.

## Data Availability

Data files, software file, Supporting Information file and README file available on online repository Dryad using reviewer link https://datadryad.org/stash/share/FjF3Y3mj6oMpL864XXu6A2OFnZukWGhxeE5dMcotgRo.
